# Effortless Totem-Pole Converter Control Using a Power Factor Correction Peak Current-Mode Controller

**DOI:** 10.3390/s24154910

**Published:** 2024-07-29

**Authors:** Abdulazeez Alsalemi, Ahmed Massoud

**Affiliations:** Department of Electrical Engineering, Qatar University, Doha 2713, Qatar; ahmed.massoud@qu.edu.qa

**Keywords:** power factor correction, totem-pole converter, peak current-mode controller, input current sensing

## Abstract

This paper expands a recently proposed peak current-mode (PCM) control method for a power factor correction (PFC) boost converter to include the totem-pole converter and solves the controller’s compatibility problem with the totem-pole converter by proposing three input current sensing methods. Using MATLAB/Simulink 2023b, simulation experiments on a 2 kW totem-pole converter utilizing the PFC PCM controller were carried out to assess the performance of the controller with the proposed sensing methods. The findings indicate that under steady-state conditions, all three proposed sensing methods performed input current shaping successfully and yielded nearly identical THD% of about 4.4% in the input current waveform. However, it is noteworthy that method 2, referred to as the memory method, exhibited a sluggish and less robust transient response in comparison to the swift and resilient responses observed with method 1 and method 3. Additionally, the third proposed method, which involves a single current sensor positioned across the input inductor, emerged as the optimal and cost-effective sensing solution. This method achieved the same desirable attributes of fast and robust control while utilizing only a single current sensor, a notable advantage over method 1, which employs two current sensors.

## 1. Introduction

Power factor correction is a critical consideration in modern power electronics and electrical systems. Its purpose is to ensure compliance with regulations by improving the power factor and lowering the THD. Recently, totem-pole converters have emerged as a fascinating and promising development. These converters represent a significant advancement in AC/DC power conversion, offering a blend of high efficiency, reduced switching losses, and enhanced power density [1]. However, the PFC control for totem-pole converters is more complex compared to conventional PFC converters since the lack of a bridge rectifier produces many challenges in PFC control design [2]. This results in much more complex control algorithms which require advanced and expensive digital signal processors (DSPs).

Over the past five years, significant attention has been directed toward the development of advanced control methods for power factor correction, driven by the need to improve efficiency [3,4] and cut the converter’s production cost. Such research efforts to reduce costs include eliminating the current sensor using sensorless current control methods [5,6,7,8,9,10,11,12] and model predictive control (MPC) [13,14,15,16,17,18,19]. However, current shaping algorithms in sensorless control techniques could be sensitive to synchronization issues [13]. Other research tends to reduce converters’ costs by adopting advanced control algorithms with low-complexity arithmetic operations. Thus, a much more affordable, low-performance microcontroller could be used. Such methods include peak and valley current control [20], and deadbeat control [21,22]. However, in peak and valley control, both analog and digital circuits could be required, while in deadbeat control, performance is affected by distorted grid voltage, circuit parameter mismatch, and control delay [22].

Other control methods in the literature include but are not limited to average current-mode control [23,24], pulse train control [23,25], one-cycle control [26,27], synchronous rectifier control (SR) [28], dynamic evolution control [29], sliding mode control [30,31,32,33], feedforward control [34,35,36,37], dual-division-summation control (D-D-∑) [38], fuzzy logic control [39], double degree-of-freedom variable control [40], finite state machine (FSM) [41], critical conduction mode control (CRM) [42,43], phase shifting control [44], adaptive control [45,46,47], and sinusoidal input current discontinuous conduction mode control [48].

**Figure 1:**
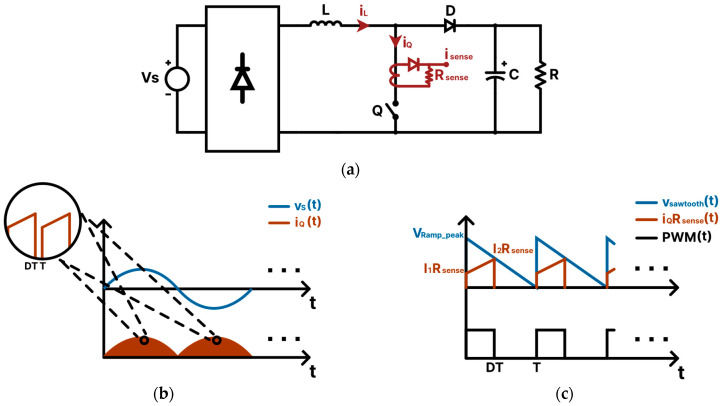
(**a**) A PFC boost converter circuit illustrating where the current sensor for the PFC PCM controller is installed. (**b**) Represents the controlled switch current waveform, while (**c**) illustrates the PWM waveform generated by the PFC peak current-mode control method.

One of the simplest methods to control PFC converters is using peak current-mode control, which compares the peak input current value with the grid voltage waveform. However, this method results in a poor power factor since the average source current must be controlled instead [49]. Recently, a promising new peak current-mode control technique suitable for PFC converters has been proposed to control a PFC boost converter [49]. This control method compares the peak current to a negative ramp sawtooth waveform to generate the PWM signal for the PFC converter switches, as illustrated in Figure 1c. The peak value of the sawtooth waveform is calculated through a simple set of equations to determine the correct compensation needed to convert the peak current value into the desired average current value. This method can achieve a unity power factor and low THD while preserving the simplicity of the algorithm. Moreover, the control realization of this controller is simple since the sawtooth signal comparison function exists in many DSPs [49]. In this paper, the PFC PCM controller is applied and adapted to the totem-pole converter. The main contributions of this paper can be summarized as follows:

- Expanding the application and the math of the PFC PCM control method to the totem-pole converter;
- Proposing three input current sensing methods for adapting the PCM controller on the totem-pole converter;
- Comparing and assessing the performance of each proposed sensing method while simulating the PCM controller on the totem-pole converter.

The rest of the paper is structured as follows. The PCM control methodology is explained in Section 2. Moreover, the compatibility of the PCM controller to the totem-pole converter is checked and the necessary equations to run the controller are obtained in Section 3. In the same section, three input current sensing methods are proposed to solve the totem-pole converter compatibility problem. In Section 4, the totem-pole converter is simulated with the PCM controller using the proposed sensing methods for performance assessment, and the results are discussed. Lastly, a conclusion that summarizes the results is found in Section 5.

## 2. PFC Peak Current-Mode Controller

**Figure 2:**
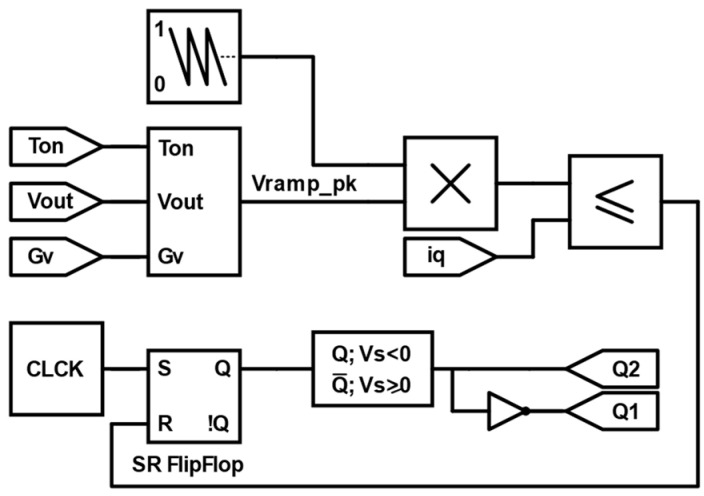
PFC peak current-mode control Simulink block diagram used in the PFC boost converter.

This section explains the operational principles and the control equations for the PFC PCM controller. As an essential prerequisite, the PFC PCM controller compatibility with any PFC converter lies in the condition that the sensed input current must exhibit a positive ramp throughout the entire grid cycle. For instance, for the PFC boost converter shown in Figure 1a, the sensed switch current $i_{Q}\left(t\right)$ satisfies the aforementioned condition since it always has a positive slope in both the positive and negative cycles of the grid voltage, as illustrated in Figure 1b. As for the operation, Figure 1c illustrates the operation of the PFC PCM controller on a boost converter [49]. The sensed current $i_{Q}\left(t\right)$, which represents the current flowing through the converter switch Q, is compared to a sawtooth function $v_{sawtooth}\left(t\right)$ with a negative ramp. The PWM signal turns on as long as $v_{sawtooth}\left(t\right)$ is greater than $i_{Q}\left(t\right)$, and the PWM on-time is determined by the peak value of $v_{sawtooth}\left(t\right)$, which is calculated such that the source average input current follows the sinusoidal grid voltage waveform, thus achieving a unity power factor and low THD. Figure 2 represents the PFC PCM control block diagram for the boost converter.

As illustrated in Figure 1c, at every switching cycle, the peak value of $v_{sawtooth}\left(t\right)$ is determined by finding the point where $v_{sawtooth}\left(t\right)$ intersects with $i_{Q}\left(t\right)$ at time DT, where T is the time period and D is the duty ratio, which can be written as:

(1) $$i_{Q}\left(DT\right)R_{sense}=v_{sawtooth}\left(DT\right),$$

where $R_{sense}$ is the sensing resistance used to measure $i_{Q}\left(t\right)$. The sawtooth equation $v_{sawtooth}\left(t\right)$ within one switching cycle is:

(2) $$v_{sawtooth}\left(t\right)=\left(1-\frac{t}{T}\right)V_{Ramp_{peak}}\left(n\right),$$

where $\left(n-1\right)T\leq t\leq nT$ and n is the switching cycle period number. As shown in Figure 1c, when $i_{Q}\left(t\right)$ intersects with $v_{sawtooth}\left(t\right)$ at DT, $i_{Q}\left(t\right)$ becomes:

(3) $$i_{Q}\left(DT\right)=I_{2}R_{sense},$$

where $I_{2}$ is the peak value of $i_{Q}\left(t\right)$ which can be written as:

(4) $$I_{2}=I_{L}+\Delta i_{L}/2,$$

where $I_{L}$ is the average input inductor current, and $\Delta i_{L}$ is the input inductor current ripple. Substituting (2), (3) and (4) into (1) results in:

(5) $${V_{Ramp}}_{peak}\left(n\right)=\frac{I_{L}+\Delta i_{L}/2}{1-D}R_{sense}.$$

Equation (5) serves as the general equation for determining the peak value of $v_{sawtooth}\left(t\right)$ throughout each cycle. Apart from the switch’s on-time $T_{on}$, which we will delve into further in Section 3, these calculations stand as the sole computations required to perform input current shaping. An additional benefit of this control strategy is that tuning is unnecessary, as all parameters in Equation (5) can be determined. Thus, introducing this control method to totem-pole converters possesses the potential to reduce their overall cost, as a less powerful DSP unit could be used. However, to ensure the compatibility of the control algorithm on the totem-pole converter or any PFC converter, Equation (5) must be validated before integration into the control system. This can be done by showing that the average input inductor current is following the grid voltage waveform $v_{s}\left(t\right)$, as shown below:

(6) $$I_{L}\left(t\right)=Gv_{s}\left(t\right),$$

where G is a constant value. To summarize, below are the steps to determine and validate ${V_{Ramp}}_{peak}$ for PFC converters.

1. Understand the operation of the PFC converter;
2. Find the PFC converter’s main parameters (e.g., $I_{L}$, $\Delta i_{L}$, and D);
3. Calculate ${V_{Ramp}}_{peak}$ using Equation (5);
4. Check if the calculated value in step c satisfies Equation (6).

## 3. Applying the PFC PCM Controller to the Totem-Pole Converter

In this section, the calculation and validation steps for ${V_{Ramp}}_{peak}$ of the totem pole converter are explained in more detail. For interested readers, the calculations made to check if the PCM controller is compatible with the PFC buck–boost, the SEPIC, and the Cuk converters can be found in Appendix A. The control equations for the PFC boost converter are not covered since detailed work has been already published in [49].

### 3.1. Determining ${V_{Ramp}}_{peak}$ for the Totem-Pole Converter

**Figure 3:**
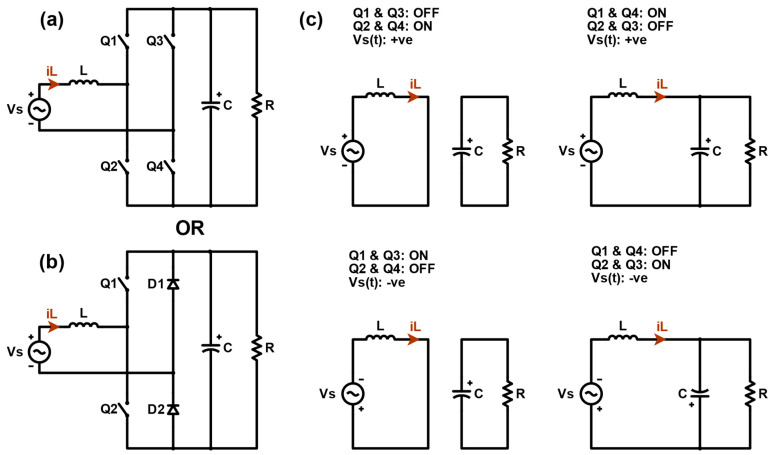
Totem-pole converter circuit with low frequency rectification using (**a**) switches (Q3 and Q4), and (**b**) diodes (D1 and D2). (**c**) illustrates their operation in CCM assuming Q3 and Q4 controlled to behave similar to diodes.

In this paper, the totem-pole converter operates in continuous conduction mode (CCM) with four operational modes during the positive and negative cycle of the AC grid voltage, as illustrated in Figure 3, where Q1 and Q2 are high-speed switches (e.g., SiC MOSFETs, GaNFETs, etc.), whereas Q3 and Q4 are low-speed switches (e.g., Si MOSFETs, IGBT, etc.). Q1 and Q2 operate at high-switching frequencies with both functions of boosting and rectifying, while the low-frequency switches are responsible for rectifying the grid voltage [50]. Thus, Q3 and Q4 in Figure 3a could be replaced with the diodes D1 and D2 demonstrated in Figure 3b without causing any changes to the PFC PCM controller.

To calculate ${V_{Ramp}}_{peak}$, the totem-pole converter’s main parameters must be found. This can be done by analyzing the converter at every operational mode [51]. However, it is sufficient to analyze the converter within the positive cycle, since the totem-pole controller ensures that it operates symmetrically with respect to the positive and negative halves of the AC input waveform. Thus, the analysis for the positive AC line cycle $V_{s}$ is as follows:

When Q1 and Q3 are open, while Q2 and Q4 are closed:

(7) $$L\frac{di_{L}}{dt}=V_{s},$$

(8) $$C\frac{dv_{c}}{dt}=-\frac{v_{C}}{R}.$$

When Q1 and Q4 are closed, while Q2 and Q3 are open:

(9) $$L\frac{di_{L}}{dt}=V_{s},$$

(10) $$C\frac{dv_{c}}{dt}=-\frac{v_{C}}{R}.$$

The change in the inductor current and the capacitor voltage while Q2 and Q4 are closed is found by modifying (7) and (8), respectively [51].

(11) $$\Delta i_{L}=\frac{V_{s}DT}{L},$$

(12) $$\Delta v_{C}=-\frac{V_{c}DT}{RC},$$

Alternatively, Equations (11) and (12) can also be computed while Q2 and Q4 are open:

(13) $$\Delta i_{L}=\frac{1}{L}\left(Vs-V_{C}\right)\left(1-D\right)T,$$

(14) $$\Delta v_{C}=\frac{1}{C}\left(i_{L}-\frac{V_{c}}{R}\right)\left(1-D\right)T.$$

In one switching cycle, the total change in the inductor current and the capacitor voltage is zero [51]. Therefore, equating (11) and (13) to zero and (12) and (14) determine the inductor current and the capacitor voltage equations.

(15) $$v_{c}=\frac{V_{s}}{1-D},$$

(16) $$I_{L}=\frac{V_{c}}{R\left(1-D\right)}.$$

By rearranging (15), the duty ratio D is obtained.

(17) $$D=\frac{V_{c}-V_{s}}{V_{c}}.$$

Now that the totem-pole converter’s main parameters are found, ${V_{Ramp}}_{peak}$ equation can be found by substituting (11) and (16) into (5)

(18) $$\frac{V_{Ramp_{peak}}}{R_{sense}}=\frac{1}{1-D}\left(\frac{v_{c}}{R\left(1-D\right)}+\frac{V_{s}DT}{2L}\right).$$

Equation (18) can be simplified further to

(19) $$\frac{V_{Ramp_{peak}}}{R_{sense}}=\left(G_{v}+\frac{T_{on}}{2L}\right)v_{c},$$

where the output voltage loop is defined as follows:

(20) $$G_{v}=\frac{1}{R{\left(1-D\right)}^{2}}.$$

It is worth noting that there is no need to calculate the value of $G_{v}$ in (20) since the output voltage loop control for the totem-pole converter can be relied on to give the correct value. Moreover, since the value of $T_{on}$ is almost equal in two consecutive switching cycles, its value can be calculated from the previous cycle [49].

### 3.2. Validating ${V_{Ramp}}_{peak}$ for the Totem-Pole Converter

Equation (19) can be validated by checking if (6) is satisfied. From Figure 1c, the inductor current rises from $I_{1}$ to $I_{2}$ during $T_{on}$.

(21) $$\Delta i_{L}=I_{2}-I_{1}=\frac{V_{s}DT}{L}.$$

Furthermore, the average inductor current is determined as

(22) $$I_{L_{avg}}=\frac{I_{1}+I_{2}}{2}.$$

By substituting (21) with (22), we obtain

(23) $$I_{avg}=I_{2}-\frac{V_{s}T_{on}}{2L}.$$

From Figure 1c, we observe that

(24) $$\frac{V_{RAMP_{peak}}}{I_{2}R_{sense}}=\frac{T}{T_{off}},$$

and the input–output relationship of the totem-pole converter can be written as

(25) $$\frac{T_{off}}{T}=\frac{V_{s}}{V_{c}}.$$

Substituting (24) into (25):

(26) $$I_{2}=\frac{V_{RAMP_{peak}}}{R_{sense}}\cdot \frac{V_{s}}{v_{c}}.$$

Substituting (29) and (26) into (23):

(27) $$I_{L_{avg}}=G_{v}V_{s}.$$

Since $G_{v}$ is constant at steady-state operation, $I_{L_{avg}}$ follows the source voltage waveform. Therefore, this control method could achieve a unity power factor.

### 3.3. Totem-Pole Converter’s Sensing Challenges and Proposed Solutions

Accurate and reliable current sensing is crucial in AC/DC power electronics converters, especially in those designed for high-power and high-frequency operation as in the totem-pole converter. These challenges arise from factors such as accuracy, bandwidth, isolation requirements, temperature stability, and the ability to effectively measure both AC and DC currents. The three current sensing methods: shunt, current transformer (CT), and hall effect (HE), are the commonly used current sensors in power electronics applications. Each offers unique advantages and considerations. Thus, careful consideration is necessary when choosing the current sensor. Table 1 presents a summarized performance comparison of the shunt, the current transformer, and the hall effect sensor, along with their weaknesses. Shunt sensing provides high accuracy ranging from 0.1% to 2% [52], but it requires careful design for safety as it is not inherently isolated [53]. It can also cause higher power losses than CT and HE current sensors, which could be significant in high-efficiency power converters. Conversely, CT sensing offers excellent isolation, making it safer. It also has lower power losses than the shunt sensor. However, it cannot measure DC currents [53]. It also suffers from saturation due to hardware limitations, which reduces the effective measurable current range. Hall effect sensing, on the other hand, is non-invasive and provides isolated measurements. It is also capable of measuring DC currents, which CTs cannot do, but at the expense of higher costs [54], higher temperature drift [52], and higher EMI susceptibility [53].

**Figure 4:**
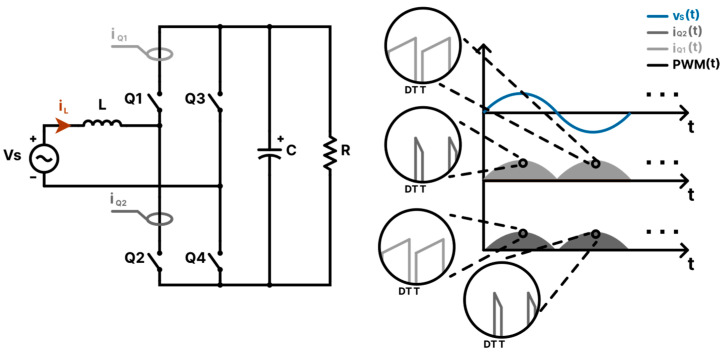
Illustrating the nature of the switch current $I_{Q1}$ and $I_{Q2}$ waveforms.

**Figure 5:**
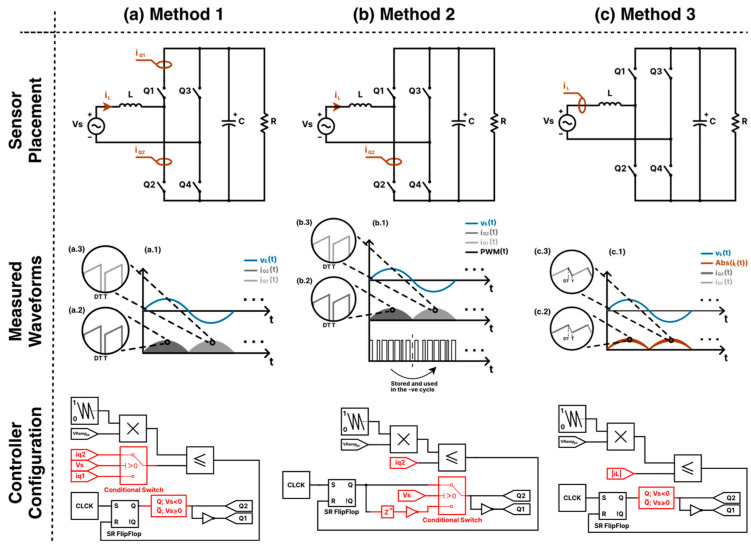
Illustration of the three proposed sensing methods for controlling the input current of the totem-pole converter. The figure includes the current sensor placement on the totem-pole circuit for each method, the waveforms demonstrating the controller’s operation, and the Simulink controller block diagram. Where subfigures (a.1,b.1,c.1) represent the plots of the grid voltage and the sensed current waveforms corresponding to sensing method 1, 2, and 3, respectively. Their zoomed-in sensed current waveforms, showing two switching periods, are illustrated respectively in subfigures (a.2,b.2,c.2) for the positive half cycles of the grid waveforms, while the negative half cycles are shown in subfigures (a.3,b.3,c.3).

Sensor placement also poses a significant challenge for integrating the PFC PCM controller into the totem-pole converter. As shown in Figure 4, it is not possible to apply the exact PFC PCM control algorithm to the totem-pole converter by placing a current sensor on a single switch, as with the PFC boost converter. This is because, unlike the boost converter, the current across any totem-pole switch $Q_{n}$ within its switching period does not have a positive ramp in both the positive and the negative grid cycles. As mentioned earlier in Section 2, it is necessary for the measured input current to have a positive ramp across the full grid cycle for the PCM controller to be compatible with the PFC converter [49]. To mitigate this problem, a modification to the control technique should be considered. Figure 5 shows the proposed sensor placement methods of the current sensors along with a demonstration of how the input current waveform is measured and the controller configuration for each method.

#### 3.3.1. Method 1: Sensing the Current across the Two Switches

Method 1 is an adaptation to the sensing method used for the PFC boost converter controller. As shown in Figure 5a, there are two current sensors. One is placed across the switch Q2 to measure the current for the positive half cycle of the grid, while the other current sensor is placed across the switch Q1 to measure the negative cycle of the grid. As suggested in [49], CT sensors can be utilized to measure the current through the two switches. Placing shunt sensors is indeed feasible; however, it comes with the cost of potentially higher power losses. HE sensors are not favored due to their restricted bandwidth compared to shunts and CTs, high-temperature drift, and elevated costs. Since this method is an adaptation of the method used in the PFC boost converter, it is expected to get a high-quality input current source with low THD values. However, from the economical perspective, utilizing a single input current sensor is preferable.

#### 3.3.2. Method 2: The Memory Method

To use a single current sensor, method 2 relies on storing the PWM signal that controls the switches within the positive half cycle, and once the negative cycle begins, it releases the stored PWM signal after inverting it. This is possible because the PWM signal required to control the input current at the positive half cycle is the inverted version of the signal. After all, switch Q1 and Q2 reverse roles in the negative cycle for proper current rectification. Figure 5b illustrates how the control method can be implemented. The PWM signal is stored in the form of a delay function ($z^{d}$) that delays the PWM signal for a half cycle. The parameter d in the delay function can be calculated as follows:

(28) $$d=\frac{T_{Vs}}{2\cdot T_{s}},$$

where $T_{Vs}$ is the grid voltage period, and $T_{s}$ is the sampling time the system is using to run the controller. Regarding the current sensor type, method 2 shares the same preferences as method 1, namely the current transformer.

#### 3.3.3. Method 3: Sensing the Inductor Current $i_{L}$

As shown in Figure 5(c.1–c.3), the input inductor current waveform of the totem-pole converter is the combination of both $i_{Q1}\left(t\right)$ and $i_{Q2}\left(t\right)$, which means a single current sensor can be placed across the input inductor for proper PCM controller integration without using two current sensors, as in method 1, or sacrificing memory resources, as in method 2. This is possible because the inductor current waveform starts at every switching cycle with a positive ramp current across the full grid cycle. In this control method, since CTs cannot measure the DC component of the inductor current, they are deemed unsuitable for this application. Consequently, a shunt current sensor is utilized instead.

In the next section, the three proposed sensing methods are simulated on the totem-pole converter using the PFC PCM controller and compared to find the best sensing method.

## 4. Simulation Results and Discussion

In this section, we present the results of simulating a 2 kW totem-pole converter utilizing the PFC PCM controller. The primary focus of our investigation involved the comparison of the three proposed current sensing methods employed on the PFC controller, and how it affects the THD of the input current and the robustness of the inner-loop controller against load disturbances. For simplification purposes, the totem-pole converter with diode line rectification was simulated. However, the controller remained unchanged when the diodes were replaced with semiconductor switches under the condition that these switches were controlled to behave like a diode. Moreover, it should be emphasized that the outer-loop controller, which regulated the output voltage to 600 V, fell outside the scope of this study. Thus, the outer-loop controller was a simple PI controller which was simply tuned using the trial-and-error approach, ensuring that its bandwidth was slower than that of the inner-loop controller [55], yet faster than twice the frequency of the grid voltage. The controller parameters for the outer-loop controller were kept unchanged across all the simulation experiments.

**Figure 6:**
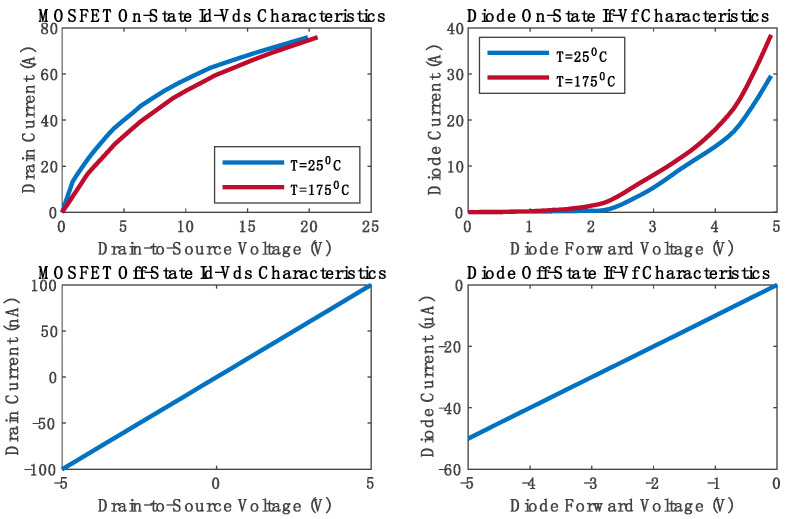
The characteristics of the AIMW120R080M1 SiC MOSFETs used in the simulation.

In this study, MATLAB/Simulink 2023b served as the primary tool for conducting the simulation experiments. For enhanced model accuracy, Simscape blocks were utilized to model physically the totem-pole converter circuit. The simulation parameters utilized for all experiments are detailed in Table 2. A simulation step time of 0.1 microseconds was employed to ensure precise capturing of the circuit’s dynamic behavior. Notably, Simscape blocks facilitated the incorporation of commercially available SiC MOSFET characteristics, enhancing the fidelity of the simulations. Thus, based on the totem-pole rated current along with the current and voltage stresses across the switches, the Infineon (Munich, Germany) AIMW120R080M1 SiC MOSFET was selected and its parameters were integrated into the Simscape MOSFET model, as outlined in Table 3. The Simscape MOSFET model considers the on-state drain current and drain-to-source voltage characteristics and the internal diode characteristics. It also considers all the SiC MOSFET’s switching losses. Figure 6 illustrates the SiC MOSFET characteristics used in our simulation experiments, which closely correspond to the specifications provided in the datasheet, affirming the accuracy and reliability of our simulation methodology. The Simulink simulation files for the three sensing methods can be found in Supplementary Material Simulations S1–S3. As demonstrated in Figure 5a–c, the controller was implemented in Simulink as follows:

1. $V_{Ramp_{peak}}$ was calculated and multiplied by a unity negative-ramp sawtooth function where its frequency determined the switching frequency of the totem-pole converter. The on-time $T_{on}$ was calculated from the previous switching cycle. $G_{v}$ was obtained from the outer voltage loop, which was selected to be a PI controller.
2. The scaled sawtooth signal was then compared to the sensed current of the respective sensing method.
3. The SR flipflop was set at the beginning of every switching cycle as long as the measured current was less than the scaled sawtooth signal. The output of the SR flipflop Q gave the PWM signal for switch Q2 and the complementary signal for switch Q1 within the positive half cycle of the grid voltage. In the negative half cycle, and as we previously discussed, Q1 and Q2 functions were inverted. Thus, their PWM signals were inverted.

**Figure 7:**
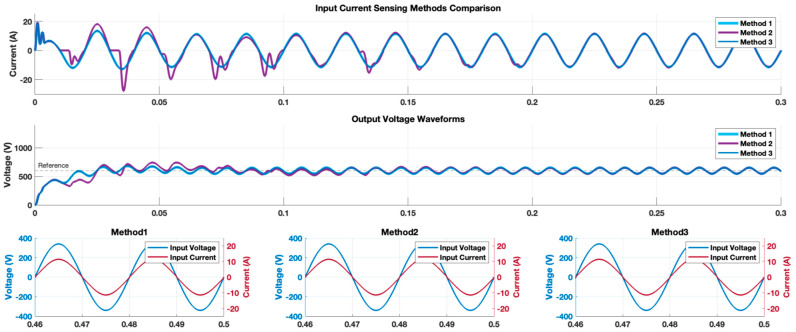
Comparing the simulated totem-pole converter input current and output voltage waveforms produced by the three proposed sensing methods. A zoomed waveform capture of the last two waveforms of the input grid voltage and current are illustrated as well.

As for the first experiment, the totem-pole converter, employing each of the proposed current sensing methods for PFC control, was simulated to assess the startup response. Figure 7 shows the simulated totem-pole converter waveforms produced by the three proposed sensing methods. The displayed waveforms include the grid current and the output voltage, along with an expanded view of two cycles of both the grid voltage and the grid current at steady state, provided for a closer inspection. Method 1 and method 3 had similar responses in which the input current had a distortion at the first half cycle, but quickly followed the grid voltage sinusoidal waveform. This was also true for the output voltage response. However, although the controller for method 2 appeared to run properly in the positive half cycle of the grid voltage waveform, the negative half cycle of the input current was highly distorted. This distortion diminished with time until it reached the same level produced by methods 1 and 3 at steady state.

**Figure 8:**
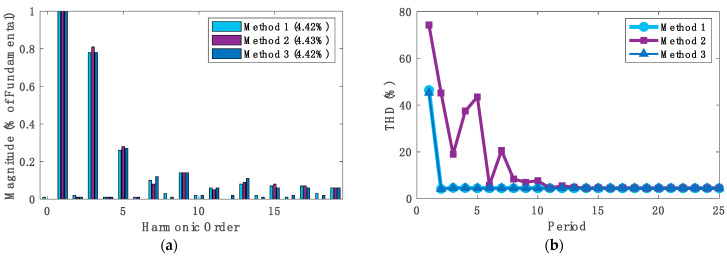
(**a**) FFT analysis of the input current waveform for each method used captured at the 25th cycle and (**b**) the THD values for the input current of the totem-pole converter at every cycle for the first 25th cycles.

Figure 8a represents the fast Fourier-transform (FFT) analysis of the input current waveform for each sensing method captured at the 25th cycle. The THD percentage of the input current waveforms, which was in the range of 4.43–4.42%, showed how a similar power quality could be obtained at steady-state using any of the proposed sensing methods, but the time to reach this low THD value varied. Figure 8b reports the THD values for the input current of the totem-pole converter at every cycle for the first 25th cycles (refer to Supplementary Material Spreadsheet S1). The figure shows that the THD values for methods 1 and 3 closely followed a similar trend over time, where at the first cycle, the THD value was around 46%. Then, the THD value decreased dramatically to about 4.1% in both methods in the second cycle, converging rapidly to the power quality standards. Finally, the THD for both methods 1 and 3 continued at low values, settling at around 4.4%. In contrast, method 2 had a significantly higher THD value in the first cycle, and its values continued to fluctuate until it settled in the 11th cycle, which was 81% more time when compared to methods 1 and 3. It is worth noting that we define the THD settling time here as the number of grid cycles required for the input current to reach and remain within 10% of the steady-state THD value.

As for how fast the system was simulated employing each sensing method, Simulink’s simulation execution time of one grid cycle for each method was measured. To reduce the results’ error caused by the fluctuating CPU performance due to uncontrollable conditions, the experiments were repeated three times, and the results were averaged (refer to Supplementary Material Spreadsheet S2). The simulation was executed on MATLAB 2023b and using a Lenovo ThinkPad T480 laptop with an i7-8550U CPU and 16GB RAM, ranking the speed from the fastest to the slowest. The results reveal that method 1 was the fastest with an execution time of 7.76 s, closely followed by method 3 at 8.53 s, and ultimately, method 2, which exhibited the longest execution time in the sequence, taking 91.45 s to complete. The reason why method 2 took significantly more execution time was that, as per Equation (28) and Table 2, the delay function stored 100,000 values of the PWM signal from the positive grid cycle interval to control the input current in the negative cycle. This significantly slowed down the control algorithm. The performance assessment values for the aforementioned experiments are reported in Table 4.

**Figure 9:**
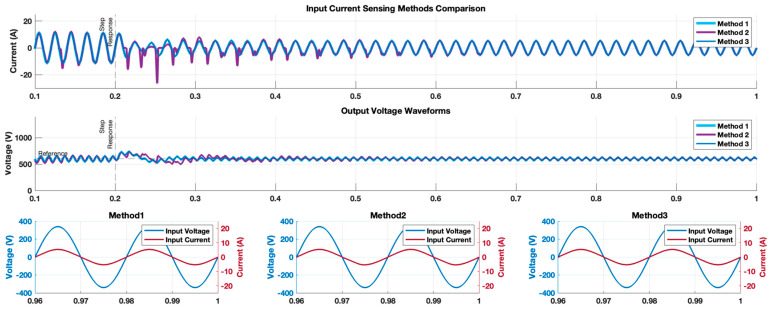
Totem-pole converter waveforms after running a load disturbance test from 2 kW to 1 kW at 0.2 s to compare the three proposed sensing methods.

To assess how the proposed sensing methods stand against external disturbances, a load disturbance test was conducted on the totem-pole converter at 0.2 s, during which the load transitioned from 2 kW to 1 kW. Figure 9 shows the simulated totem-pole converter waveforms produced by the three proposed sensing methods. The input current waveforms showed a similar behavior to that in Figure 7 at the moment of the load change: methods 1 and 3 responded robustly and quickly to the load change, while method 2 had difficulties maintaining a stable state. Both method 1 and method 3 reached steady-state operation within approximately 0.06 s following the load change. In contrast, method 2 required approximately 0.5 s to return to the original THD% value.

Following the analysis of the two simulation experiments conducted, we derived the following insights:

- The integration of sensing method 1 or 3 into the PFC PCM controller yielded nearly identical input current and output voltage waveforms, leading to similar behavior and THD% value that conformed to the power quality standards. The primary distinction lay in the sensor count: method 3 employed a single switch, in contrast to method 1’s two switches. This offered a notable advantage to method 3, enhancing controller simplicity and cost-effectiveness, because managing a single sensor was easier than attempting to merge the current sensing data of two sensors for PFC control. However, this came at the cost of using a shunt current sensor, which normally has higher power losses. Thus, by proposing several sensing techniques that all satisfy the power quality standards, designers are afforded the flexibility to select the most appropriate solution based on their specific needs and design constraints.
- The concept of method 2 is based on the assumption that the totem-pole converter has an identical operation in the two half cycles. However, an examination of the waveforms in Figure 7 and Figure 9 reveals a discrepancy: while the input current waveform remained sinusoidal during the positive half cycle when employing method 2, it became severely distorted in the negative half. This indicates that the totem-pole operation was not identical in both half cycles. Moreover, the practice of storing the PWM control signal from the positive half and applying it to the negative half led to this distortion and a sluggish response to sudden load variations. Additionally, method 2 required substantial memory resources, resulting in simulation times that were more than 10 times longer than those of methods 1 and 3. Therefore, given these operational challenges, method 2 is not suitable for practical use without modification.

Overall, the three sensing methods proposed open up new opportunities by making the PFC PCM controller compatible with the totem-pole converter, cutting their cost and simplifying the controller’s architecture, which has profound implications for the design and manufacturing processes. Furthermore, this controller eliminates the need for tuning, simplifying its use relative to alternative PFC input current controllers designed for the totem-pole converter. These enhancements will likely lead to increased adoption and broader application of the totem-pole converter technology in the industry. It is now clear that the memory method (method 2) is not an appropriate solution for the peak current mode controller of the totem-pole converter due to its poor input current transient response and lengthy execution time. Moreover, the results imply that method 3, which employs a single current sensor, could be used to apply the PFC PCM controller to reduce costs without compromising on the power quality and stability of using two current sensors, as in method 1.

## 5. Conclusions

This paper explores the application of the PFC PCM controller in totem-pole converters. While totem-pole converters offer significant advancements in AC/DC power conversion, their PFC control is inherently more complex due to the absence of a bridge rectifier. The proposed PCM controller, initially designed for PFC boost converters, was extended to totem-pole converters. The study addresses the challenges of controller compatibility through the introduction of three input current sensing methods. Simulation experiments conducted using MATLAB/Simulink revealed that all three sensing methods resulted in nearly identical THD of about 4.42% under steady-state conditions. The outcomes highlight that method 3, which employed a single current sensor across the input inductor, was the optimal and cost-effective sensing solution. It provided fast and robust control with the advantage of utilizing only a single current sensor. This method’s simplicity and efficiency offer a significant advantage over the other methods evaluated. Method 1 had a similar performance to method 3. However, it lacks the simplicity and cost benefits associated with method 3, making it a less preferred option when considering the balance between complexity and efficiency. Lastly, the integration of sensing method 2 into the totem-pole’s PCM controller was found to be impractical due to its poor performance. This research contributes to the understanding and implementation of efficient PFC control in totem-pole converters, paving the way for enhanced performance and practical applications in power electronics and energy-efficient technologies.

## Figures and Tables

**Table 1:** Current sensors performance comparison [52,53,54].

	Shunt	Current Transformer	Hall Effect
Accuracy	0.1–2%	0.1–1%	0.5–5%
Isolation	Not inherently isolated	Isolated	Isolated
Bandwidth	kHz–MHz	kHz–MHz	kHz
Cost	low	Moderate	high
Circuit Invasion	Invasive	Non-invasive	Non-invasive
Temperature Drift (ppm/K)	25–300	<100	50–1000
Power Loss	mW–W	mW	mW
DC Capability	yes	No	Yes
Weaknesses	Requires careful design due to its non-isolated nature; high power losses	Current saturation; inability to measure DC current	Susceptible to EMI; high temperature drift; low bandwidth

**Table 2:** Totem-pole converter simulation parameters.

$\mathrm{Grid}\;\mathrm{Voltage}\;(V_{RMS}$)	Grid Frequency (Hz)	Input Inductor (mH)	Output Capacitor (μF)	Output Voltage (V)	Power Rating (W)	Switching Frequency (kHz)	Simulation Step/Sampling Time (μs)
240	50	1	100	600	2000	100	0.1

**Table 3:** The details and parameters for the selected SiC MOSFET in the simulation.

Switch Type	Part Number	Manufacturer	$\mathrm{Drain}-\mathrm{Source}\;\mathrm{Voltage}\;(V_{DS})$	$\mathrm{Typical}\;\mathrm{DC}\;\mathrm{Drain}\;\mathrm{Current}\;(I_{D})$	$\mathrm{Drain}-\mathrm{Source}\;\mathrm{on}-\mathrm{State}\;\mathrm{Resistance}\;\left(R_{DS\left(on\right)}\right)\;$
Silicon Carbide MOSFET	AIMW120R080M1	Infineon	1200 V	33 A	80 mΩ

**Table 4:** Performance assessment data for the previous experiment.

	Method #1	Method #2	Method #3
Simulink execution time for one period (s)	7.76	91.45	8.53
THD at steady state (%)	4.42	4.43	4.42
THD settling time (cycles)	2	11	2
Number of current sensors	2	1	1

**Figure A1:**
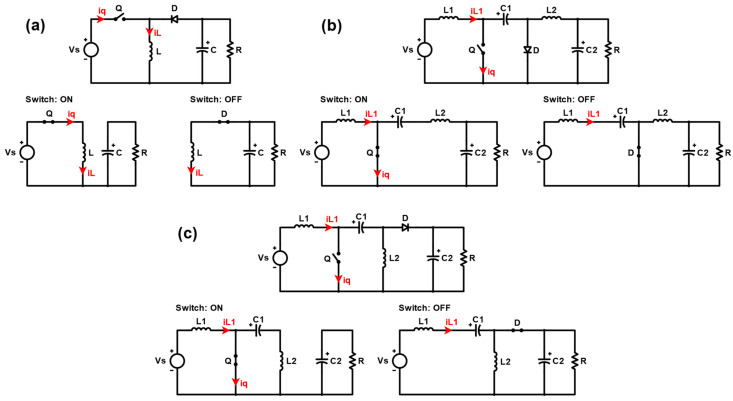
The equivalent circuit operation for (**a**) the buck–boost converter, (**b**) the Cuk converter, and (**c**) the SEPIC converter.

## Data Availability

Data are contained within the article and Supplementary Materials.

## Supplementary Materials

The following supporting information can be downloaded at: https://www.mdpi.com/article/10.3390/s24154910/s1, Spreadsheet S1: THD of the three methods for the first 25 periods.xlsx; Spreadsheet S2: Simulink execution time for each sensing method.xlsx; Simulation S1: Method1.slx; Simulation S2: Method2.slx; Simulation S3: Method3.slx.

## Appendix A

### Appendix A.1. Buck–Boost Converter

Figure A1a shows the equivalent circuit for buck–boost converter operation when the switch is closed and open respectively. Analyzing the PFC buck–boost converter at steady-state operation, the following equations were obtained [51]:

(A1)
$$I_{L}=\frac{V_{s}D}{{\left(1-D\right)}^{2}R},$$

(A2)
$$\Delta i_{L}=\frac{V_{s}DT}{L},$$

(A3)
$$V_{out}=-\left(\frac{D}{1-D}\right)V_{s},$$

(A4)
$$D=\frac{\left|V_{out}\right|}{V_{s}+\left|V_{out}\right|}.$$

Substituting (A1)–(A4) into (5)

(A5) $$\frac{V_{Ramp}}{R_{sense}}=\left(G_{v}-\frac{T}{2L}\right)V_{out},$$

where

(A6) $$G_{v}=-\frac{1}{{\left(1-D\right)}^{2}R}.$$

Applying the same steps used in verifying the controller of the totem-pole converter:

(A7) $$I_{avg}=DG_{v}V_{s}$$

Since D in (A7) is a variable value at steady-state operation, the control method cannot be used in the PFC buck–boost converter.

### Appendix A.2. Cuk Converter

Figure A1b shows the equivalent circuit for Cuk converter operation when the switch is closed and open, respectively. Analyzing the PFC Cuk converter at steady-state operation, the following equations were obtained [51]:

(A8) $$V_{out}=-\left(\frac{D}{1-D}\right)V_{s},$$

(A9) $$I_{L1}=-\left(\frac{D}{1-D}\right)\frac{V_{C2}}{R},\;$$

(A10) $$\Delta i_{L1}=\frac{V_{s}DT}{L_{1}},$$

(A11) $$D=\frac{V_{out}}{V_{out}+V_{s}}.$$

Substituting (A8)–(A11) into (5)

(A12) $$\frac{V_{Ramp}}{R_{sense}}=\left(G_{v}-\frac{T}{2L_{1}}\right)$$

(A13) $$G_{v}=-\frac{D}{{\left(1-D\right)}^{2}R}$$

Applying the same steps used in verifying the controller of the totem-pole converter:

(A14) $$I_{avg}=\left(1-D\right)G_{v}V_{s}$$

Since D in (A14) is a variable value at steady-state operation, the control method cannot be used in the PFC Cuk converter.

### Appendix A.3. SEPIC Converter

Figure A1c shows the equivalent circuit for SEPIC converter operation when the switch is closed and open, respectively. Analyzing the PFC SEPIC converter at steady-state operation, the following equations were obtained [51]:

(A15) $$V_{out}=V_{s}\left(\frac{D}{1-D}\right),$$

(A16) $$D=\frac{V_{out}}{V_{out}+V_{s}},$$

(A17) $$I_{L1}=\frac{V_{out}^{2}}{V_{s}R},$$

(A18) $$\Delta i_{L1}=\frac{V_{s}DT}{L_{1}}.$$

Substituting (A15)–(A18) into (5)

(A19) $$\frac{V_{Ramp}}{R_{sense}}=\left(G_{v}+\frac{T}{2L_{1}}\right)V_{C2}.$$

Applying the same steps used in verifying the controller of the totem-pole converter:

(A20) $$I_{avg}=DG_{v}V_{s}$$

Since D in (A20) is a variable value at steady-state operation, the control method cannot be used in the PFC SEPIC converter.
